# Supplementary material for: Are carbon emissions trading and green financial instruments synergistic? -Comprehensive quantitative research based on content analysis
Source: PLoS One. 2024 Mar 7;19(3):e0298601. doi: 10.1371/journal.pone.0298601 (PMC10919601; doi:10.1371/journal.pone.0298601)
Supplement: S1 Data — (ZIP) [file pone.0298601.s001.zip › Data/地级市Z_F工作报告.zip!/▒▒╛⌐/└┤╫╘╣1⁄2╓┌║┼í╛┬φ┐╦╩2╛▌═°í┐_2017.docx]

——2017年1月14日在北京市第十四届人民代表大会第五次会议上

北京市代市长 蔡奇

各位代表：

　　现在，我代表北京市人民政府，向大会报告政府工作，请予审议，并请市政协各位委员提出意见。

　　一、2016年工作回顾

　　过去一年，在党中央、国务院的亲切关怀下，在中共北京市委的领导下，在市人大及其常委会的监督支持下，我们深入贯彻落实习近平总书记视察北京重要讲话精神，认真落实《京津冀协同发展规划纲要》，牢固树立创新、协调、绿色、开放、共享的发展理念，坚持稳中求进工作总基调，坚持首都城市战略定位，着力推进供给侧结构性改革，加快疏功能、转方式、治环境、补短板、促协同，较好地完成了全年主要目标任务，实现了“十三五”良好开局。初步预计，全市地区生产总值比上年增长6.7%左右，居民消费价格上涨1.4%，城镇登记失业率1.41%。一般公共预算收入增长7.5%，城乡居民人均可支配收入实际增长6.7%左右。万元地区生产总值能耗、水耗和二氧化碳排放分别下降4%以上、3%左右和5%左右，细颗粒物年均浓度下降9.9%。

　　(一)京津冀协同发展取得新成效。非首都功能疏解有序推进。严格实施新增产业禁止和限制目录，累计不予办理登记业务1.64万件。关停退出一般制造业和污染企业335家，疏解各类商品交易市场117家。北京城市学院、北京建筑大学等向郊区疏解取得阶段性进展。天坛医院新院、同仁医院亦庄院区二期项目积极推进。

　　城市副中心建设进展顺利。完成副中心总体城市设计和6个重点地区详细城市设计。行政办公区抓紧建设。统筹实施基础设施、生态环境等5大领域350项重点工程，已开工106项。环球主题公园项目开工建设。

　　重点领域率先突破取得新成果。京张铁路、京沈客专北京段按计划推进，京唐城际北京段实现开工，京台高速北京段建成通车。实施京津冀大气污染防治强化措施，牵头建立京津冀及周边地区水污染防治协作机制。支持张承生态功能区建设，京冀生态水源保护林新增10万亩，京津风沙源治理二期工程完成林业建设任务19.7万亩。曹妃甸示范区北京项目开工35个，天津滨海-中关村科技园起步区开工建设，保定·中关村创新中心入驻的北京企业和机构达到45家，落户沧州的北京生物医药企业达到53家，北京现代四工厂竣工投产。新机场主体工程和配套设施建设全面开工，临空经济区规划获得批复。

　　协同创新共同体积极构建。落实京津冀系统推进全面创新改革试验方案，出台中关村国家自主创新示范区京津冀协同创新共同体建设行动计划。推动建设张北云计算基地等创新载体，打造了一批跨区域的创新创业服务平台。

　　冬奥会、冬残奥会筹办工作扎实开展。组建筹办工作机构，编制完成总体计划及任务分工方案，启动场馆规划设计工作，国家速滑馆设计方案征集进展顺利。广泛开展系列冬奥文化体育活动，群众参与冰雪运动的热情持续升温。

　　世园会园区建设全面启动，交通、市政等配套基础设施建设有序推进，园区招展工作取得积极进展。

　　扎实做好对口支援和帮扶协作，投入资金42.65亿元，实施援助项目661个。建立结对帮扶机制，深化京冀对口帮扶合作。

　　(二)改革开放深入推进。全面落实深化改革各项任务，一些重点领域改革取得突破性进展。持续深化“放管服”改革，取消77项行政审批事项，终结非行政许可审批，取消调整74项基层开具的各类证明。全面推行“五证合一”，企业登记注册时间缩短至2到4个工作日。公共服务类建设项目投资审批改革试点效果明显。市政务服务中心投资项目在线审批监管平台上线运行。公布市政府部门权力清单和责任清单。

　　积极推进重点领域改革，出台促进民间投资的政策措施，持续推进政府和社会资本合作示范项目。实施新一版政府定价目录，形成水电气热非居民区域差别化价格体系。出台国资国企改革实施方案，推进15项改革试点，退出“僵尸企业”55户，市属企业利润保持稳定增长。

　　深入开展服务业扩大开放综合试点，完成试点任务的80%，形成可复制推广的8项体制机制创新，催生了10种新业态。服务贸易总额增长10%以上，“双自主”企业出口占比进一步提高。实际利用外资130亿美元,境外直接投资155亿美元。成功举办第四届京交会。

　　(三)经济发展新动能持续培育。制定供给侧结构性改革实施方案，“三去一降一补”任务全面落实。提前完成国家下达化解煤炭产能180万吨的年度任务。“营改增”改革试点全面推开，新增减税175.7亿元。降低社会保险费率，减轻企业负担50亿元。

　　实施加强全国科技创新中心建设总体方案，制定科研项目和经费管理28条改革措施，落实外籍人才出入境管理20条政策，实施“互联网+”行动计划。大众创业、万众创新持续活跃，新技术、新产品、新业态、新模式不断涌现，新设科技型企业增长22.4%。中关村示范区总收入增长12%以上，全市技术合同成交额增长14.1%，科技对经济增长的贡献率超过60%。

　　高端产业发展态势良好，金融、信息、科技服务业对经济增长的支撑作用明显，服务业占地区生产总值的比重超过80%。文化创意产业活力不断增强，企业总收入增长8%以上。实施《中国制造2025》北京行动纲要，现代制造业增加值增长10%以上。

　　(四)城市治理迈出坚实步伐。出台全面深化改革提升城市规划建设管理水平的意见，调整组建市规划国土委、市城市管理委。开展城市总体规划编制工作，实现城市总体规划和土地利用规划两图合一。

　　强化城市治理工作，拆除违法建筑超过3000万平方米，清理地下空间1400多处，整治违法群租房1.56万间，清理无证无照经营3.3万户。开展背街小巷环境整治和区域环境提升，完成城六区100公里架空线入地。制定户籍制度改革实施意见，实行居住证制度，出台积分落户办法。全市常住人口增量、增速继续保持“双下降”，城六区常住人口实现了由增到减的“拐点”。

　　持续抓好大气污染防治，完成8480蒸吨燃煤锅炉改造、663个村煤改清洁能源，压减燃煤200万吨、总量降至1000万吨以下，淘汰老旧机动车44万辆，清理整治“散乱污”企业4470家。加强环境污染治理，新增生活垃圾日处理能力5350吨，垃圾资源化率达到56%；污水处理率达到90%，再生水利用量10亿立方米。建成南水北调通州支线及配套水厂，中线工程全年调水超过11亿立方米，密云水库蓄水量突破16亿立方米。实现平原地区生态林管护全覆盖，完善山区生态林补偿政策。新增城市绿地408公顷，完成造林19万亩，全市林木绿化率达到59.3%。

　　扎实推进交通缓堵工作，广渠路二期等重点道路建成通车，地铁16号线北段顺利开通，轨道交通新增运营里程20公里，总里程达到574公里，新增公交专用道100公里，治理自行车道300公里，中心城区绿色出行比例达到71%。

　　(五)城乡发展一体化水平进一步提高。积极推进大兴区农村集体经营性建设用地改革试点，首宗地块入市交易。制定深化农村改革综合性实施方案，基本完成村级集体经济产权制度改革，国有林场改革全面开展。北京长城国家公园体制试点区实施方案获得批复。制定实施通州、房山、大兴国家新型城镇化综合试点方案，统筹推进42个重点小城镇建设。“一绿”地区城市化建设第一批试点乡完成90%腾退任务，农民全部整建制转居。

　　落实调结构转方式发展高效节水农业的意见，全市粮田面积调减到110万亩。实施农村基础设施“六网”改造提升工程，完成农民住宅抗震节能改造7.6万户。对低收入村和低收入农户实施精准帮扶措施，低收入农户人均可支配收入增速高于全市农民。

　　(六)社会民生不断改善。28件重要民生实事全部落实。积极做好就业创业工作，城镇新增就业42.8万人。有序推进城乡居民基本医疗保险制度整合，门诊、住院最高支付数额和大病保险待遇实现统一。机关事业单位养老保险制度改革基本完成。开展因病致贫家庭医疗救助，实施困难残疾人生活补贴和重度残疾人护理补贴。深入落实居家养老服务条例，出台支持居家养老服务发展十条政策，建设街乡镇养老照料中心53个、社区养老服务驿站150家。建设提升便民商业网点1700个。出台房地产调控8条措施，促进房地产市场平稳健康发展。保障性住房新开工5.6万套、竣工6.4万套，完成3.9万户棚户区改造任务。利用疏解、拆违腾退空间，建设一批公园绿地、文体中心和停车场等公共服务设施，改善了人居环境。

　　基本公共服务水平稳步提升。出台深化中高考考试招生制度改革实施方案，推进中小学校长教师交流轮岗，义务教育免试就近入学比例进一步提高。实施第二期学前教育三年行动计划，新增学前教育学位1.5万个。出台城市公立医院综合改革实施方案，推进药品阳光采购，市属医院实行非急诊全面预约挂号。健全分级诊疗制度，建立基层医疗机构4类慢病双向转诊基本标准，新增10个区域医联体。创建国家公共文化服务体系示范区，落实全民健身实施计划，群众文化体育事业繁荣发展。

　　加强和创新社会治理，有序推进“三网融合”示范点建设。完成村委会换届选举工作。积极促进妇女儿童事业发展，加强民族、宗教、对台、侨务工作。深化国防动员和双拥共建，军民融合发展取得新成效。扎实做好安全生产工作，实施食品药品安全三年行动计划，安全生产和食品药品安全形势总体稳定。深入排查化解矛盾纠纷，积极推进社会治安防控体系转型升级，强化反恐防恐能力建设，严厉打击各类违法犯罪行为，保持了首都和谐稳定的良好局面。

　　过去一年，政府自身建设取得了新的进展。全面深入贯彻党的十八届六中全会精神，切实增强“四个意识”特别是核心意识、看齐意识，严守政治纪律和政治规矩。认真开展“两学一做”学习教育。全面执行市人大及其常委会的决议和决定，自觉接受人大工作监督、法律监督和政协民主监督，认真听取各民主党派、工商联、无党派人士和人民团体意见，共办理市人大代表议案4件、建议1049件，办理市政协提案938件。认真做好政府立法工作，提请市人大常委会审议地方性法规4项，制定、修改、废止政府规章11项。严格落实中央八项规定精神，加大“四风”问题监督检查力度，开展“为官不为”、“为官乱为”问题专项治理。加强行政监察和审计监督，强化绩效管理，开展行政问责。落实“一岗双责”，推进政府系统廉政建设和反腐败斗争，着力解决发生在群众身边的不正之风和腐败问题，严肃查处了一批违纪违法案件。

　　各位代表，过去一年取得的成绩，是党中央、国务院坚强领导的结果，是中共北京市委带领全市人民团结奋斗的结果。在此，我谨代表北京市人民政府，向全市人民，向全体人大代表、政协委员，向各民主党派、各人民团体和各界人士，向中央和国家机关各部门各单位、各兄弟省区市，向驻京解放军和武警部队官兵，向所有关心支持首都建设的港澳台同胞、海外侨胞和国际友人，表示衷心的感谢！

　　与此同时，我们清醒地认识到，我市经济社会发展还存在不少困难和问题，政府工作也存在一些差距和不足。主要是：人口资源环境矛盾依然突出，空气污染、交通拥堵、垃圾污水治理任务艰巨，治理“大城市病”还要下很大功夫；稳增长压力依然较大，科技创新资源优势尚未充分发挥，新兴产业支撑不足；社会民生事业仍有许多群众不满意的地方，优质公共服务供给总量不足与配置不均衡并存；城市管理体制机制还需要不断完善，精细化水平亟待提高；政府职能转变还不到位，监管方式相对滞后，有些部门的服务意识不强、服务效率不高；一些政府工作人员不作为、乱作为，顶风违纪违法行为时有发生，等等。对此，我们将本着对党和人民高度负责的态度，直面问题，勇于担当，在今后工作中认真加以解决。

　　二、2017年主要任务

　　2017年是实施“十三五”规划、率先全面建成小康社会、建设国际一流的和谐宜居之都的关键一年，也是落实《京津冀协同发展规划纲要》的重要一年。首都发展面临着难得的历史机遇，也存在着诸多挑战。中共北京市委十一届十二次全会已经对今年工作作出了全面部署。最重要的是深入学习贯彻习近平总书记系列重要讲话精神和治国理政新理念新思想新战略，全面贯彻落实习近平总书记视察北京重要讲话精神，这是做好各项工作的根本遵循；“四个中心”是首都城市战略定位，要牢固确立，切实履行好“四个服务”职责；首善标准是一贯追求，要始终坚持，力争各项工作做得更好。就当前和今后一个时期而言，要紧紧围绕以疏解非首都功能为重点的京津冀协同发展战略来发力。疏解非首都功能，实际上就是供给侧结构性改革，就是调结构、转方式，就是腾笼换鸟，就是提升城市发展质量，就是改善人居环境，就是缓解人口资源环境的突出矛盾，就是更好地履行作为国家首都的职责。必须坚持以疏解非首都功能为工作导向，以提升首都核心功能、建设国际一流的和谐宜居之都为目标方向，把疏解与提升有机结合起来，在疏解功能中谋发展。我们坚信，有党中央、国务院的亲切关怀，有中共北京市委的坚强领导，有全市人民的团结奋斗，就一定能够在已有工作基础上坚韧不拔、攻坚克难，乘势而上、不辱使命。

　　今年政府工作的总体要求是：全面贯彻党的十八大、十八届三中、四中、五中、六中全会和中央经济工作会议精神，统筹推进“五位一体”总体布局和协调推进“四个全面”战略布局，坚持以习近平总书记视察北京重要讲话精神为根本遵循，坚持稳中求进工作总基调，牢固树立和贯彻落实新发展理念，适应把握引领经济发展新常态，坚持以提高质量和效益为中心，坚持以推进供给侧结构性改革为主线，加快疏功能、转方式、治环境、补短板、促协同，全面做好稳增长、促改革、调结构、惠民生、防风险各项工作，促进首都经济平稳健康发展和社会和谐稳定，以优异成绩迎接党的十九大胜利召开。

　　全市经济社会发展的主要预期目标是：地区生产总值增长6.5%左右；一般公共预算收入增长6.5%；居民消费价格涨幅控制在3.5%左右；城镇登记失业率控制在3%以内；城乡居民人均可支配收入增长与经济增长同步；万元地区生产总值能耗、二氧化碳排放分别下降3.5%、4%，万元地区生产总值水耗下降3%左右，细颗粒物年均浓度力争控制在每立方米60微克左右。

　　(一)落实首都城市战略定位，建设国际一流的和谐宜居之都，必须坚定有序疏解非首都功能，全力推动京津冀协同发展。

　　坚持疏存量、控增量。严格落实新编制的城市总体规划，严格管控生态红线和城市开发边界。修订并落实污染行业淘汰退出目录，关停退出500家以上一般制造业和污染企业。全面清理整治镇村产业小区和工业大院。加大疏解批发市场力度，完成动物园地区、大红门地区、天意、永外城等批发市场的撤并升级和外迁，基本完成官园、万通、雅宝路地区等批发市场的调整疏解和升级改造。加快部分市属高校、医院疏解步伐，全面推进既定项目的落实，抓好中国人民大学、北京电影学院等高校新校区建设，加快建设友谊医院顺义院区，实现天坛医院新院试运行。严格执行新增产业禁止和限制目录，严控城六区人口规模和开发强度。落实腾退空间管理和使用意见，建立新增建设用地与疏解腾退土地挂钩机制。

　　推动三个重点领域率先取得突破。积极推动京津冀地区城际铁路网和首都地区环线高速规划建设，实现京秦高速全线竣工，抓好轨道交通平谷线建设，促进京津冀交通一体化。加强区域生态环境保护，持续推进京津风沙源治理工程，完成10万亩京冀生态水源保护林、4万亩京津保造林绿化任务。强化区域水污染防治协作，推动永定河、潮白河、北运河等跨省市河流环境整治和生态修复。做好产业对接协作，引导产业项目向曹妃甸示范区等地转移集聚，推动天津滨海-中关村科技园打造特色鲜明的创新服务平台，支持张承生态功能区绿色产业发展。扎实推进新机场外围市政交通项目，启动实施临空经济区规划。

　　高水平建设城市副中心。坚持城市副中心的功能定位，以新的理念编制各项规划设计，严格规划执行。抓紧行政办公区和配套设施建设，年底四大市级机关和相关市属行政部门率先启动搬迁。完善副中心内部路网系统，建设广渠路东延段并开展沿线环境整治。推进一批优质教育医疗项目落地。建设环球主题公园。基本消除通州区黑臭水体，实现副中心现有建成区污水全收集全处理。实施海绵城市试点，大力推进森林湿地和公园绿地建设。深入挖掘以运河为核心的历史文化资源，塑造鲜明的城市特色。坚持建管并举，努力提高城市副中心管理水平。

　　扎实推进冬奥会、冬残奥会和世园会筹办工作。编制完成赛事基础规划和赛事交付计划，建立可持续管理、无障碍服务等工作体系。新建竞赛场馆和相关基础设施全面开工，积极推进现有竞赛场馆改造工作。启动市场开发，发布冬奥会和冬残奥会会徽。加强赛事组织管理人才培养，实施科技冬奥行动计划，推动冰雪运动进校园、进社区，普及冰雪运动和冬奥知识。全面开工建设世园会园区展馆、世园村基础设施，基本完成公共景观建设，开工建设部分展园，推进国际国内招展工作。启动2020年世界休闲大会筹备工作。

　　认真做好对口支援和区域合作。聚焦精准扶贫、精准脱贫，抓住改善民生、特色产业等重点领域，开展对河北省16个区县的对口帮扶，认真做好援藏、援疆、援青等对口支援工作，完成京蒙对口帮扶、京沈对口合作及南水北调对口协作年度任务。

　　(二)落实首都城市战略定位，建设国际一流的和谐宜居之都，必须大力推进供给侧结构性改革，促进首都经济提质增效升级。

　　深入调整供给结构。着力抓好“三去一降一补”，全面完成国家下达的年度任务。坚决退出低端无效供给，支持首钢集团化解外埠产能，退出处理危险废物以外的全部水泥产能。开展亏损企业专项治理，支持分类处置50户以上“僵尸企业”。研究制定促消费政策，实施消费品标准升级和质量提升规划，大力发展健康养老、文化体育、休闲旅游等新兴消费，进一步提高服务消费比重。规划建设一批特色旅游村镇和旅游休闲度假项目，推进昌平、延庆、平谷、怀柔、门头沟全域旅游示范区创建工作。聚焦重点促投资，在京津冀协同发展、高精尖产业、生态环境、基础设施、民生改善等领域，实施230项市级重点工程。落实促进民间投资27条具体措施，推动重点项目落地。

　　加快推进关键性改革。深入推进“放管服”改革，继续取消下放行政审批事项，清理审批中介服务和基层各类证明。推进公共服务类建设项目投资审批改革试点。所有投资项目实行一站式审批。开展全程电子化登记和市场主体简易注销登记试点。加强信用管理和诚信体系建设。探索建立跨部门、跨领域“双随机、一公开”监管机制。大力推进政府和社会资本合作，重点抓好11个国家级示范项目。积极防范化解金融风险。深化国企国资改革，研究推出职业经理人、员工持股等改革试点。以混合所有制改革为突破口，加大企业调整重组力度。进一步放宽市场准入，促进民营经济发展。

　　着力推动产业高端化发展。落实加快生产性服务业发展的实施意见，巩固提升金融、科技、信息、商务服务等优势产业，大力发展节能环保服务、计量标准、检验检测认证、人力资源等新兴产业，加快奥体文化商务园、新首钢高端产业综合服务区建设。支持传统优势企业实施绿色制造和智能制造技术改造，加快机器人创新产业基地、集成电路产业园、新能源智能汽车产业园建设，推动创建“中国制造2025”示范区。完善促进总部经济发展的政策措施，优化提升总部经济。

　　深入推进农村改革发展。在全市开展农村集体经营性建设用地乡镇统筹利用试点，基本完成承包经营权确权登记颁证，推进土地征收制度改革。深化国有林场改革，基本完成国家集体林业综合改革试验示范区建设任务。深入推进国家新型城镇化综合试点工作，培育发展功能性特色小城镇。高质量完成第三次全国农业普查任务。大力推进农业供给侧结构性改革，继续调减退出高耗水种养农业，着力发展现代种业、休闲农业、乡村旅游等都市型现代农业，增加绿色优质农产品和生态产品供给。抓好北京鲜活农产品流通中心主体工程。推进300个美丽乡村建设，实施6万户农民住宅抗震节能改造，全面完成第三轮山区搬迁计划。聚焦低收入农户增收，大力推进“六个一批”精准帮扶，努力改善低收入群众生活。

　　提高开放型经济发展水平。深化服务业扩大开放综合试点，完成国家已批复的试点任务，抓紧推出试点深化方案和新一轮开放措施，促进服务业转型升级和服务贸易发展。打造更有国际竞争力的投资环境，促进外资外贸稳中有进。落实我市参与建设“一带一路”实施方案，引导企业有序“走出去”，深化与中蒙俄经济走廊沿线重点城市合作，推动建设一批境外产能合作项目。深化京港澳互利合作。做好对台和侨务工作。

　　(三)落实首都城市战略定位，建设国际一流的和谐宜居之都，必须下大气力治理“大城市病”，提升城市可持续发展水平。

　　综合施策加强人口调控。实施“疏解整治促提升”专项行动，把疏解非首都功能、城市综合治理专项行动与人口调控紧密挂钩，形成支撑，确保取得明显成效。坚决遏制新增违法建设，拆除违法建筑4000万平方米以上。集中开展直管公房违规转租转借和商改住清理整治，持续整治群租房和出租大院，完成地下空间三年综合整治任务。严厉打击开墙打洞、占道经营、无证无照经营等行为。推进城乡结合部地区综合整治，完成100个市级挂账重点地区整治任务。全面启动“一绿”地区城市化建设，推进“二绿”地区城乡一体化建设。落实户籍制度改革措施，完善积分落户标准和政策，做好人口服务管理。

　　加大力度治理大气污染。深入研究把握大气污染成因和防治规律，进一步提高治理的实效性。全面实施环境保护督察，层层压实责任，推动清洁空气行动计划各项任务落到实处。继续大力压减燃煤，完成700个村煤改清洁能源，实现城六区和南部平原地区基本“无煤化”，实施4000蒸吨燃煤锅炉清洁能源改造，工业企业燃煤设施“清零”，全面淘汰10蒸吨及以下燃煤锅炉，采暖季结束后华能北京热电厂燃煤机组停机备用，全年压减燃煤30%、总量降至700万吨以内。抓好重型柴油车管控，推动实施六环路重型柴油车限行，公交、环卫等行业新增重型柴油车全部安装颗粒捕集器。在城六区、北京经济技术开发区、通州区划定区域实施低排区管控，不符合国Ⅲ标准的非道路移动机械一律禁止使用。供应第六阶段成品油，淘汰老旧机动车30万辆，两年以上出租车全部更换三元催化器。进一步推广新能源汽车，新建3000个公用充电桩。加大工业治污减排力度，完成1万蒸吨燃气锅炉低氮改造，加快实施燕化低氮改造、挥发性有机物泄漏检测修复等治理工程，清理整治2570家“散乱污”企业。严格控制扬尘污染，全面落实施工单位责任，开展渣土车专项整治。严格执行排放标准，增加环保执法编制，组建环保警察队伍，强化环境监管执法，严厉惩处偷排超排行为。严格落实新修订的空气重污染应急预案，加强区域大气污染联防联控，完善预警会商和应急联动机制，及时发布相关信息，积极回应市民关切，动员全社会共同参与，切实做好空气重污染应对。

　　实施新一轮缓堵专项行动计划。坚持公交优先战略，强化公交服务，新开和优化调整40条公交线路，完成600公里自行车道和步道整治，中心城区绿色出行比例提高到72%。加强交通设施建设，开工建设2条轨道交通新线，年内增加运营里程30公里。推进兴延、延崇等高速公路建设，抓紧建设西外大街西延、姚家园路、丽泽路等城市快速路。实施100项疏堵工程，推进城六区次支路建设，畅通道路微循环。开工建设北苑北、苹果园等综合交通枢纽。推进智慧交通建设，集中开展交通秩序整治行动，遏制因乱致堵。增加居住区停车设施，加强路侧停车管理，在城六区建设12处停车管理示范区。深化出租车行业改革，促进巡游车、网约车融合发展。

　　着力改善生态环境质量。建立生态文明建设目标评价和考核体系。实施新一轮污水处理设施建设三年行动计划，突出抓好污水管线和农村分散处理设施建设，城六区建成区基本完成污水收集管网铺设。严格落实“河长制”，实施水环境区域补偿机制，改善国家和市级监测断面水质。建设生态清洁小流域29条，完成57条段黑臭水体治理任务，全面消除建成区黑臭水体。建成运行6项垃圾处理设施，推进生活垃圾源头减量和分类回收利用，生活垃圾资源化率达到57%。深入开展农业面源污染治理，全面禁止秸秆焚烧，完成100家规模化养殖场污染治理。实施土壤污染防治工作方案，做好工业企业原厂区污染土壤治理，开展废弃矿山修复工作。推进延庆、密云、怀柔、平谷生态文明先行示范区建设，提高门头沟生态发展水平。新增造林16万亩、城市绿地600公顷，建设14处城市休闲公园和郊野公园，恢复和增加湿地2200公顷。

　　加强城市精细化管理。强化市级统筹协调，完成组建区级城市综合管理机构，深化城管执法体制改革，完善联合执法机制，推动执法重心下移。全面推进城市管理、社会服务、社会治安、城管综合执法等多网融合，推广“北京通”，实施一批智慧惠民工程，积极建设智慧城市。深入开展综合治理和环境提升，改善中心城区市容市貌。持续推进城区架空线入地。抓好地下综合管廊建设。全面建设节水型社会，制定出台推进海绵城市建设的实施意见，大力推进城六区自备井置换，完成汛后水毁修复工程。加强防灾减灾能力建设，强化资源能源日常保障。继续做好南水北调工程运行管理，加快建设大兴支线等配套工程，编制完成并抓紧实施后续规划。推进陕京四线等天然气工程建设，建立完善热电气常态化联调联供机制，确保城市运行平稳有序。

　　(四)落实首都城市战略定位，建设国际一流的和谐宜居之都，必须深入实施创新驱动发展战略，加强全国科技创新中心建设。

　　全国科技创新中心是建设世界科技强国的重要支撑，是首都发展的新引擎。坚持以“三大科学城”建设为主平台，以高校院所、创新型企业为主力军，以重大科研项目和科学工程为抓手，以深化改革为动力，加快建设全国科技创新中心。建立全国科技创新中心建设目标体系，加强年度考核评估，确保各项任务落到实处。

　　建设“三大科学城”。加强基础设施建设，完善公共服务配套，全力服务保障国家实验室在京布局，积极配合国家科技重大专项在京实施并争取承担更多重大科技任务，主动对接国家科技创新面向2030年的重大项目和重大工程，超前布局脑科学、人工智能、生物技术、石墨烯和第三代半导体等基础前沿研究，着力提升原始创新能力，打造世界知名科学中心。进一步拓展和优化中关村科学城发展空间，积极承接国家实验室、“两机”专项等重大项目，在前沿科学技术创新、成果转化、双创平台建设和环境服务提升等方面实施一批项目，发挥中关村科学城的引领、辐射和带动作用。全面落实怀柔科学城发展规划，细化完善各专项规划，推进综合性国家科学中心建设，开工建设地球系统数值模拟等2个国家重大科技基础设施和材料基因组研究等一批前沿交叉研究平台，推进高能同步辐射光源等3个国家重大科技基础设施立项前期工作，努力打造科学综合实力新高地。实施未来科技城行动计划，持续引进“千人计划”等高端创新创业人才，建设高水平企业研发中心，实施10项关键技术攻关，加快20项重大科技成果转移转化，提升科技城发展活力。

　　深化科技体制机制改革。发挥中关村改革试验田作用，在激发科技人员积极性、推动技术与资本结合等方面探索新的改革举措。建立重大项目统筹落地机制，促进一区多园高端化、差异化发展。全面落实科研项目和经费管理28条政策措施，赋予科研单位和人员更多自主权，推动中央在京科研单位适用北京的创新激励政策。推进中关村人才管理改革试验区建设，实施外籍人才申请在华永久居留积分评估制度，实行以增加知识价值为导向的分配政策。在新材料、智能机器人等领域，引进、支持全球顶尖科学家及创新团队。加快国家科技金融创新中心建设，深化投贷联动、外汇管理改革等试点，完善创业投资引导机制，吸引更多社会资本投入科技创新。深入推进中关村“双创”综合改革试点和海淀区国家“双创”示范基地建设，依托高校院所、企业建立专业化“双创”平台，引导众创空间、创新型孵化器高端化发展。加强知识产权运用、保护和标准化工作，推进中关村国家知识产权服务业集聚发展示范区建设，提升北京品牌的影响力。

　　加快构建协同创新共同体。深入推进京津冀全面创新改革试验，推动区域创新政策交叉覆盖，在健全区域知识产权联动服务机制、开展跨区域联合监管等方面实现突破。聚焦区域节能减排、污染防治等发展需求，联合开展关键技术研发和应用示范。培育新能源汽车等重点行业联盟，为区域内企业提供技术支持和科技服务。搭建企业为主体的创新载体，支持企业集团在京津冀合理布局研发、孵化、制造、售后等环节，形成协同创新产业链，推进共建科技园区和产业基地建设。

　　(五)落实首都城市战略定位，建设国际一流的和谐宜居之都，必须坚持社会主义先进文化前进方向，加强全国文化中心建设。

　　培育和践行社会主义核心价值观。拓展百姓宣讲、中国梦365个故事等宣传品牌影响力，用中国梦和社会主义核心价值观凝聚共识，坚定文化自信，推动全社会形成共同的理想信念和价值追求。加强爱国主义教育，办好纪念全民族抗战爆发80周年相关活动。开展“2017北京榜样”大型主题活动，评选表彰第六届首都道德模范，做好先进典型、时代楷模、最美人物、身边好人和优秀志愿者的选树宣传。统筹推进文明城区、文明村镇、文明单位、文明家庭和文明校园创建工作，进一步提升市民素质和城市文明程度。

　　完善公共文化服务。推动首都公共文化服务示范区创建，提高文化综合服务效能。加强乡镇、街道综合文化中心建设，推进数字化图书馆、公共电子阅览室建设，提高基层公共文化设施水平。健全公共图书、文化活动、公益演出三大配送体系，把更多公共文化产品送到基层，倡导全民阅读。创新百姓周末大舞台等演出形式和内容，举办2万场次首都市民系列文化活动。落实繁荣发展社会主义文艺的意见，充分发挥文化艺术基金引导作用，扶持创作重大革命历史题材、京味文化、传统文化等主题作品，争取产出更多文艺精品。推动戏曲传承创新，举办中国戏曲文化周系列活动。深化市属国有文化企业改革，完善国有文化企业分类监管。推动文化创意产业功能区转型升级，抓好国家文化产业创新实验区等一批重大项目建设，促进文化创意和设计服务与相关产业融合发展，办好北京国际设计周。

　　保护好历史文化名城“金名片”。做好中轴线申遗前期准备工作，推进景山公园寿皇殿建筑群修缮，推动恢复“一轴一线”魅力景观。加强旧城整体保护，推进文保区腾退疏解和有机更新，保护南锣鼓巷等历史文化街区特色，延续古都历史文脉。传承弘扬优秀民族文化和民俗文化，扶持非物质文化遗产代表性项目和传承人，支持老字号品牌传承发展。统筹推进长城、运河、西山文化带建设，加强历史文化景区整体保护和环境整治。积极保护名镇、名村和传统村落。

　　(六)落实首都城市战略定位，建设国际一流的和谐宜居之都，必须坚持以人民为中心，切实保障和改善民生。

　　积极回应群众关切，扎实办好重要民生实事。实施提高生活性服务业品质行动计划，建设提升1000个便民商业网点，推动规范化、连锁化、品牌化发展。加强高校毕业生、就业困难群体和农村转移劳动力就业帮扶，做好相关企业职工分流安置，实现城镇新增就业36万人。全面整合城乡居民基本医疗保险制度。实施精准救助，加大“救急难”力度，保障困难群众基本生活。积极做好困境儿童和留守儿童保障工作。深化残疾人社会保障和公共服务体系建设，加强残疾人融合教育，加快残疾人小康进程。完善社会化养老服务体系，大力发展居家养老，建设200家社区养老服务驿站，发展农村互助养老和志愿服务，深入推进医养结合。把握住房的居住属性，以建立购租并举的住房制度为主要方向，以政府为主提供基本保障，以市场为主满足多层次需求，金融、财税、土地、市场监管等多措并举，探索建立符合国情市情、适应市场规律的基础性制度和长效机制，促进房地产市场平稳健康发展。加大中低价位、中小套型普通商品住房供应比例，保障房建设筹集5万套、竣工6万套。棚户区改造3.6万户。完成1.5万套自住型商品住房供地。培育和规范发展住房租赁市场。加快发展装配式建筑。以城六区为重点，继续推进老旧小区综合整治，切实改善群众居住条件。

　　发展更高质量更加公平的教育。坚持把立德树人作为中心环节，把思想政治工作贯穿教育教学全过程。持续推进素质教育，完善实践育人体系。加快城乡义务教育学校标准化建设，实施乡村教师支持计划和特岗计划，深化学区制改革，支持集团化发展和九年一贯制办学，扩大优质教育资源覆盖面。大力发展学前教育，新建、改扩建一批公办幼儿园，扶持发展普惠性民办幼儿园。继续实施中招市级统筹各项举措，推动优质高中招生名额分配向一般初中校倾斜。实施特殊教育学校办学条件达标工程。开展中高职与本科教育贯通培养改革试点。深化高等学校高水平人才交叉培养，引导部分高校向应用型转变，办出特色、争创一流。

　　深入推进健康北京建设。制定实施健康北京2030规划纲要，促进卫生与健康事业改革发展。协调推进医疗、医保、医药联动改革，全面实施医药分开，调整医疗服务价格，推进复合型医保支付方式改革。大力推行药品阳光采购，完善基本药物制度，促进大医院与社区用药衔接。推进分级诊疗，加快紧密型医联体建设，引导部分二级医院向康复护理医院转型。完善基层医疗绩效考核制度，推广家庭医生签约服务，提高基层医疗服务能力。实施中医药治未病健康工程，支持中医药振兴发展。落实院前医疗急救服务条例，加强重点传染病和慢病防控。优化妇幼保健和计划生育服务，增加助产服务资源，提高儿科救治能力。认真执行新修订的北京市全民健身条例，新建专项活动场地509片，开展丰富多彩的全民健身活动。大力开展群众性赛事活动，提高竞技体育发展水平。

　　确保首都和谐稳定。开展全民国家安全教育，牢固树立总体国家安全观，切实做好维护安全稳定、促进社会和谐各项工作。加强和创新社会治理，深化社会组织管理制度改革，健全枢纽型社会组织工作体系，培育发展社区社会组织。深化街道社区管理体制改革，推进社区减负增效、社区协商，建设100个一刻钟社区服务圈。支持国防和军队建设改革，抓好全民国防教育，加强双拥共建，推动军民深度融合发展，巩固发展军政军民团结。做好人民防空工作。认真贯彻民族工作方针政策，推进民族团结进步事业发展。依法管理宗教事务，构建积极健康的宗教关系。制定安全生产领域改革发展实施方案，狠抓安全生产责任制落实，启动全市安全生产重大风险源普查，开展危险化学品正面清单编制工作和集中管理体系建设，完善隐患排查治理和安全预防控制体系，坚决遏制重特大事故发生。大力推进消防基础设施建设，提升消防综合应急救援能力。推进国家食品安全城市创建工作，强化食品药品全过程监管，让群众饮食用药安全放心。加强重大决策社会稳定风险评估，落实信访工作责任制，完善人民调解、行政调解和司法调解相融合的多元调解体系，有效排查化解社会矛盾纠纷，解决群众合理合法的利益诉求。严格落实反恐防恐各项措施，加强监测预警预防，坚决打击暴力恐怖活动。完善立体化、信息化社会治安防控体系，依法治理网络空间，防范打击电信网络诈骗犯罪，深化公安执法规范化建设，严厉打击违法犯罪行为，增强人民群众的安全感。

　　今年，党的十九大将在北京召开，“一带一路”国际合作高峰论坛也将在北京举办。做好相关服务保障工作，是中央赋予的光荣政治任务。要周密部署，精心组织，抓好重点区域的环境整治和景观提升，努力营造安定祥和的社会环境，确保重大活动服务保障工作万无一失。

　　三、切实加强政府自身建设

　　做好新一年工作，加强政府自身建设是保障。政府系统要全面深入贯彻党的十八届六中全会精神，切实增强“四个意识”特别是核心意识、看齐意识，更加自觉地在思想上政治上行动上同以习近平同志为核心的党中央保持高度一致，坚决维护党中央权威。进一步强化对权力运行的制约和监督，努力营造风清气正的政治生态。各级领导干部要带头坚定理想信念，严守政治纪律和政治规矩，切实做到忠诚干净担当，不忘初心、继续前进，为人民服务永远在路上。

　　坚持依法行政，严格按程序决策、按规矩办事，注重统筹兼顾。依法接受市人大及其常委会的工作监督和法律监督，坚决落实各项决议、决定并定期报告工作。自觉接受市政协民主监督，认真听取各民主党派、工商联、无党派人士和人民团体的意见。认真办理人大代表议案建议和政协提案，进一步提高办理质量。积极推进法治政府建设，科学编制政府立法工作计划，推动城市建设和管理等重点领域立法。完善行政执法和监督体系，推动执法责任落实。全面推行政府法律顾问制度，做好行政复议和行政应诉工作。完善政务信息公开三级责任清单，推行重大行政决策预公开制度，畅通社会监督渠道。完善公共法律服务体系，深入开展“七五”普法，进一步增强全社会法治观念。

　　大力转变工作作风，坚持密切联系群众，深入基层开展调查研究，着力解决实际问题。进一步完善并严格落实权力清单和责任清单，加快四级政务服务体系建设，大力推行“互联网+政务服务”，让企业和群众办事更方便。完善常态化督促检查工作机制，健全限期报告、情况通报、专项督查等制度，严格绩效管理考核，确保重大决策部署落实到位。对工作落实不力、效能低下、敷衍塞责、弄虚作假等行为，严肃追责问责。坚持一张蓝图绘到底，看准了的事一抓到底，落实工作一竿子插到底。督促和激励广大公务员强化责任担当，保持奋发有为的精神状态，发扬艰苦奋斗的工作作风，做出让人民群众满意的实绩。

　　坚定不移推进政府系统廉政建设和反腐败斗争。认真落实“一岗双责”，深化廉政风险防控，严格执行廉洁自律准则和纪律处分条例，自觉遵守中央八项规定精神，坚决反对“四风”，坚决把纪律和规矩挺在前面。加大审计监督力度，推进公共资金、国有资产、国有资源、领导干部经济责任审计全覆盖。坚持有腐必反、有贪必肃，加大违纪违法案件查处力度，坚决防止和纠正损害群众利益的不正之风，切实做到干部清正、政府清廉、政治清明。

　　各位代表，首都改革发展正处在关键时期，任务艰巨，责任重大。让我们更加紧密地团结在以习近平同志为核心的党中央周围，在中共北京市委的坚强领导下，团结依靠全市人民，凝心聚力，开拓进取，撸起袖子加油干，为建设国际一流的和谐宜居之都、谱写中华民族伟大复兴中国梦的北京篇章而努力奋斗，以优异成绩迎接党的十九大胜利召开！
